# From the classroom to Facebook: a modern approach for smoking education in adolescents

**DOI:** 10.1186/1617-9625-12-S1-A19

**Published:** 2014-06-06

**Authors:** Antonis A Kousoulis, Stylianos P Kympouropoulos, Dimitra K Pouli, Konstantinos P Economopoulos, Constantine I Vardavas

**Affiliations:** 1 Clinic of Social and Family Medicine, Medical School, University of Crete, Heraklion, 71003, Greece; 2 Society of Junior Doctors, Athens, 15123, Greece; 3 Department of Psychiatry, General University Hospital “ATTIKON”, Athens, Greece; 4 School of Medicine, National and Kapodistrian University of Athens, Athens, 115 27, Greece; 5 Department of Surgery, Massachusetts General Hospital, Harvard Medical School, Boston, Massachusetts, 02114, USA; 6 Center for Global Tobacco Control, Department of Social and Behavioral Sciences, Harvard School of Public Health, Boston, Massachusetts, 02114, USA

## Background

Although studies show that a large majority of smokers initiate smoking while in early adulthood, poor efficacy of school smoking-prevention programs has been noted. With the explosive rise in internet use, the imperative need to use new forms of media for educational purposes has emerged more prominently than ever before. In this paper we briefly describe the design and testing of a smoking-related social media-integrated education intervention.

## Materials and methods

We describe 5 simple steps towards a successful presentation that will lead to meaningful social media interactions: Steps 1-3 include the careful selection of the presenters and their education on general presentation techniques and classroom behaviors, as well as the topic in particular. During step 4, the presenters link up with the students using a social media platform, and provide “take-home” key points. Facebook is chosen as the most popular social networking website in which the vast majority of youth is daily active and already has accounts. Finally, in step 5, the presenters request the attendees to upload one of the take home messages as their Facebook status. The rationale is that the knowledge could, thus, reach a quite larger number of young people than the finite number of attendees.

## Results

We implemented and tested the above algorithm during a tobacco control lecture curriculum to 225 high school students in Athens, Greece, in May 2012. After the lecture, a 3-day window was provided to the attendees to connect with the presenters in Facebook and post a smoking-related sentence in their account status. Assessed 72hrs later, 32 students (14.2%) had posted a smoking-related sentence in their Facebook account, a “take-home message” that was spread to their 20,095 cumulative friends as a note on their wall via newsfeed.

## Conclusions

Our research describes a successful implementation of an educational intervention on smoking in high school students. Should an educational or community based campaign utilize a Facebook function and systematize this algorithm within a number of schools, the take-home messages heard from the always influential lips of their peers, could reach literally the entire community of adolescents and presumably lead to social sensitization through offline networks that have an online representation. This algorithm and its preliminary implementation adds to the limited existing evidence on how social media may advance tobacco control, and provides insight into a novel way of providing health information to youth, a hard to reach and vulnerable population.

